# A Customizable Model for Chronic Disease Coordination: Lessons Learned From the Coordinated Chronic Disease Program

**DOI:** 10.5888/pcd13.150509

**Published:** 2016-03-31

**Authors:** Karen Voetsch, Sonia Sequeira, Amy Holmes Chavez

**Affiliations:** Author Affiliations: Sonia Sequeira, Amy Holmes Chavez, National Center for Chronic Disease Prevention and Health Promotion, Centers for Disease Control and Prevention, Atlanta, Georgia; Oak Ridge Institute for Science and Education, Oak Ridge, Tennessee.

## Abstract

In 2012, the Centers for Disease Control and Prevention provided funding and technical assistance to all states and territories to implement the Coordinated Chronic Disease Program, marking the first time that all state health departments had federal resources to coordinate chronic disease prevention and control programs. This article describes lessons learned from this initiative and identifies key elements of a coordinated approach. We analyzed 80 programmatic documents from 21 states and conducted semistructured interviews with 7 chronic disease directors. Six overarching themes emerged: 1) focused agenda, 2) identification of functions, 3) comprehensive planning, 4) collaborative leadership and expertise, 5) managed resources, and 6) relationship building. These elements supported 4 essential activities: 1) evidence-based interventions, 2) strategic use of staff, 3) consistent communication, and 4) strong program infrastructure. On the basis of these elements and activities, we propose a conceptual model that frames overarching concepts, skills, and strategies needed to coordinate state chronic disease prevention and control programs.

## Introduction

Several factors drive the rationale for a coordinated approach to chronic disease prevention and control. Chronic diseases, which affect millions of Americans every year, are interrelated (1). Individuals have not one disease, but several comorbidities, and these largely spring from the same core risk factors: smoking, physical inactivity and poor nutrition, excessive alcohol consumption, high blood pressure, and high cholesterol (2). Risk factors and chronic diseases can be prevented or managed by many of the same strategies and interventions both at the individual and systems level (including health care interventions) and the population level (2).

Because Congress allocates most federal funding for state-based chronic disease prevention and control through categorical budget lines, programs were developed for certain diseases and risk factors. Although this practice led to strong programs and deep expertise in states and at the Centers for Disease Control and Prevention (CDC), it has also created “silos” that impede a cohesive public health agenda to chronic disease prevention and control. A singular focus on one disease can lead to competition with others for resources and attention. Programs may miss opportunities to gain efficiencies by failing to capitalize on shared strategies. And large systems that govern the public’s behavior — worksites, health care systems, community institutions, and others — are less likely to adopt a disjointed set of public health strategies promoted by separate and distinct programs.

The challenge is to coordinate these efforts and leverage opportunities and skills for greater efficiencies and health impact. An assessment of all 50 state health departments identified “improved health outcomes, common risk factors better addressed, and reduced duplication of program efforts” as the most commonly anticipated benefits of coordination (3). To support a more coordinated approach, the National Center for Chronic Disease Prevention and Health Promotion (NCCDPHP) provided resources and technical assistance to all states and territories and the District of Columbia under the Coordinated Chronic Disease Program (CCDP). States were required to address 8 broad categories (Table 1) with the intent of enhancing states’ capacity in NCCDPHP’s 4 domains: epidemiology and surveillance, environmental approaches, health care transformation, and community–clinical linkages (4). The CCDP initiative marked the first time that NCCDPHP provided resources to all state health departments explicitly for comprehensive planning, improvements in infrastructure, and capacity for coordination.

**Table 1:** Coordinated Chronic Disease Prevention Categories

Category	Description
Program management and leadership	Engage staff and stakeholders in the health department to develop new processes, functions, structures, or capacities.
Surveillance and epidemiology	Demonstrate the use of surveillance and epidemiology data to plan, implement, and evaluate programs.
Evaluation	Evaluate measurable outcomes and monitor progress toward achievement of programmatic objectives and outcomes using process, output, programmatic, and epidemiology and surveillance data and information.
State chronic disease prevention and health promotion plan	Develop or update and implement a state coordinated chronic disease prevention and health promotion plan that describes key objectives and action areas.
Organizational structure	Develop or enhance the chronic disease unit organization structure to strengthen leadership, enhance coordination and collaboration across chronic disease prevention activities, and share best practices across multiple program areas.
Collaborative processes	Develop or enhance collaborative processes with coalitions, multisector and nontraditional partners and linkages with health care systems.
Communication	Develop and implement a communication plan that describes the social and economic burden of chronic diseases, conditions, and risk factors, and chronic disease prevention and health promotion interventions.
Policy	Develop, strengthen, or intensify efforts to implement policy strategies to increase the number, reach, quality and impact of statewide, local, and organizational policies that support health and healthful behaviors.

This article summarizes the lessons learned from that experience and informs a conceptual model, which frames overarching concepts, skills, and strategies needed to coordinate state chronic disease prevention and control programs. To our knowledge, such a conceptual model has not been published in the scientific literature.

## Methods

We selected 21 states from 50 eligible state health departments by using a combination of purposive and criteria-based sampling. We queried 5 CDC program consultants who worked directly with states on coordination efforts to recommend 2 or 3 states per NCCDPHP region that had achieved all of the objectives of the CCDP program and had sustained their efforts in coordination beyond the project period. This process ensured that our sample represented geographic diversity and progress in coordination. Using a codebook, developed through inductive analysis (5) to discover patterns and themes of coordination, we analyzed approximately 80 programmatic documents from the 21 states using NVIVO 10 (QSR International). Chronic disease directors from 7 of the 21 states were selected for key informant interviews on the basis of the robustness of the data from the document review and recommendations from CDC consultants. Using a grounded theory approach, we analyzed the interviews and documents; analysis entailed line-by-line coding conducted by 2 raters who held regular discussions to resolve any discrepancies in coding. This thematic analysis informed the development of the conceptual model (Table 2), and we selected quotes from the interviews and documents that further describe each element of the model. The sources of the quotes are identified by 2 acronyms: CDDI (chronic disease director interview) or SPD (state planning document). 

**Table 2:** Data Collection Methods, Coordinated Chronic Disease Program, 2012

Data Analysis	Selection Criteria	No. of States	Data Source Description
Document review^a^	Progress in coordination and sustained activities: Queried 5 CDC program consultants who were each responsible for providing technical assistance to states through the CCDP program. • CDC program consultants recommended states that had successfully achieved CCDP objectives and sustained those activities beyond the project period. • 2 or 3 states per NCCDPHP region were included for geographic diversity	21 of 50 states and the District of Columbia	80 programmatic documents from the 21 states, including • CCDP plans • Sustainability plans • Communication plans • Evaluation plans • Critical functions assessment • Progress reports
Key informant interviews	Information-rich participants: • Robust data from document review • Recommendations from CDC program consultants	7 of 21 states	Notes from key informant interviews of 7 chronic disease directors
Conceptual model development^b^	NA	NA	Emerging themes from the document review and key informant interviews

Abbreviations: CCDP, Coordinated Chronic Disease Program; CDC, Centers for Disease Control and Prevention; NA, not available; NCCDPHP, National Center for Chronic Disease Prevention and Health Promotion.

a NCCDPHP allocated states into 9 regions (A–J) to support a regional approach to technical assistance across the Center. Region A: Connecticut, Maine, Massachusetts, New Hampshire, New York, Rhode Island, Vermont; Region B: Delaware, the District of Columbia, Maryland, New Jersey, Pennsylvania, Virginia, West Virginia; Region C: Florida, Georgia, North Carolina, South Carolina; Region D: Alabama, Kentucky, Mississippi, Tennessee; Region E: Illinois, Indiana, Michigan, Minnesota, Ohio, Wisconsin; Region F: Arkansas, Louisiana, New Mexico, Oklahoma, Texas; Region G: Iowa, Kansas, Missouri, Nebraska; Region H: Colorado, Montana, North Dakota, South Dakota, Utah, Wyoming; Region I: Arizona, California, Hawaii, Nevada; Region J: Alaska, Idaho, Oregon, Washington.

b Versions of the model were carefully examined by CDC program staff and Regional Representatives, a group of chronic disease directors who provide ongoing feedback to CDC.

## Conceptual Model Elements

**Figure 1:**
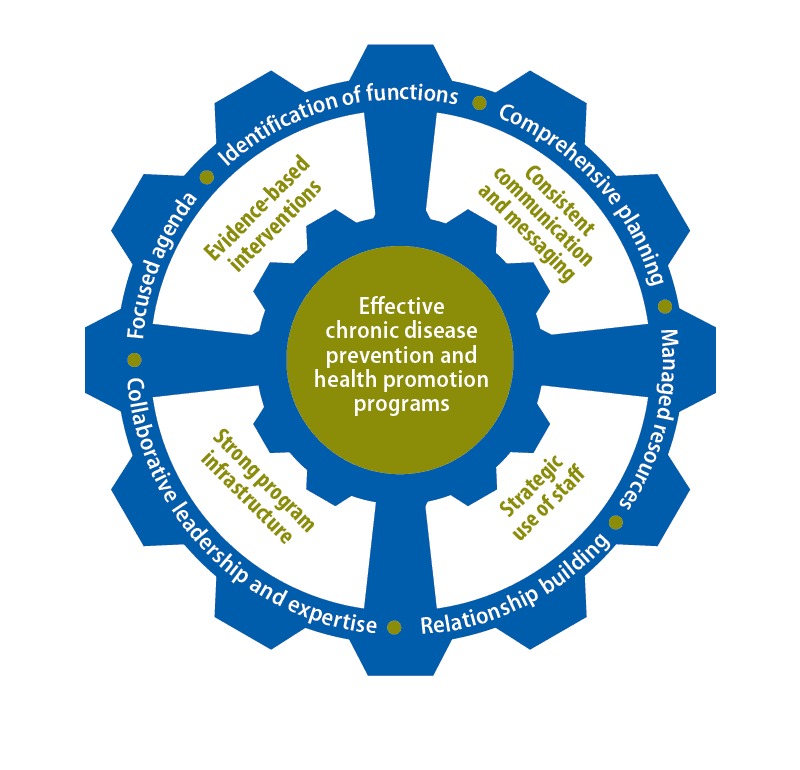
Conceptual model for chronic disease coordination.

The resulting conceptual model (Figure 1), developed by CDC in 2015, outlines the high-level functions and activities that state health departments put in place to shift to a more coordinated approach. The center of the model represents the goal: effective chronic disease and health promotion programs. Four essential activities surround this goal and are guided by the 6 components of leadership and management identified in the outer ring. 

### Leadership and management

Organizations make successful changes because their members understand the need for urgency and are motivated to change (6). “Collaborative leadership and expertise” represents high-level support for coordination as an organizational and institutional norm. State health department leaders communicated the urgency of coordination, remained open to feedback through staff engagement and group facilitation, but were also prepared to make tough decisions.

At times, this meant making unpopular decisions about program direction. There was a noticeable point when managers shifted from trying to create desire for change among employees to moving on — and that it was left up to those who were still resistant to make a decision to adapt or leave (SPD).

A “focused agenda” refers to a concise set of chronic disease prevention and control strategies with the potential for high population impact that becomes a shared vision among program leadership, staff, and partners. State health departments charted this path by developing a chronic disease state plan, which required a carefully navigated course to obtain support from varying partners and interest groups. This course resulted in renewed attention to chronic disease issues (http://wtnh.com/2014/05/28/live-healthy-connecticut-plan). An integrated chronic disease plan was one of several recommendations highlighted in a 2007 article in *Preventing Chronic Disease*, “Recommendations for Integration of Chronic Disease Programs” (7).

“Relationship building” refers to obtaining buy-in for a coordinated approach with internal and external partners and identifying new opportunities for collaboration and public health action. State health departments’ restructuring of their categorical disease programs and their partnerships led to breakthroughs with Medicaid, the US Department of Transportation, and other major systems, such as education and health care. State health department personnel connected with key decision makers at these organizations where decisions had a better chance of having population-wide impact.

It’s hard to know if [it’s because of] the coordinated approach or the time is now, but I think by talking about [chronic diseases] at a higher level, we’ve been asked to be at more tables. We are expected to be there to talk about chronic disease and not stroke specifically (CDDI).

“Comprehensive planning” is defined as the process of aligning separate chronic disease coalitions or partnerships and creating a formalized arena in which the state health department can receive input on its chronic disease program planning and implementation. Many states described a statewide chronic disease leadership team or advisory group that works strategically to align all chronic disease prevention and control resources and provides direction for public and private partners.

While it took some time to align the work of the partnership with the Division’s priorities, this alignment has enabled the state health department to strengthen its efforts, provided necessary input into its strategic direction, and created better coordination with external partners (SPD).

“Managed resources” — an element of the Component Model of Infrastructure developed by the Office on Smoking and Health at CDC — are funds or social capital that produce social benefits (8). Many state health departments described using fiscal tools for combining funds (eg, blending or braiding) or identifying new, previously untapped resources to enhance chronic disease prevention and control science and programs.

Managed resources also refers to supporting a more fluid and flexible structure aligned to meet specified goals. In the past, organizational design was often determined by mechanisms of funding rather than by outcome-oriented goals. In these new structures, staff often have a range of expertise and cross training or functional skills that are transferable to several disease or risk factor areas.

The management team conducted an organizational assessment and made several decisions: with each open position, conduct another assessment to determine needs of staff as compared to the work that must be achieved and conduct assessments of our structure to make sure it matches the needs (SPD).

The “identification of functions” refers to the practice of determining important staff functions across the chronic disease unit in areas such as epidemiology, surveillance, evaluation, communications, and policy analysis and creating efficiencies in staffing across programs. This process is in line with a recent Institute of Medicine (IOM) report and other publications that call for defining the core chronic disease competencies and capabilities that should be universally available for our public health system to work anywhere (9,10).

As a part of the organizational realignment, all job descriptions were revised to reflect consistency in scope and skill level with a more of functional descriptions concept. This provides an opportunity for staff to not only master a specific program or content area, but allows them to expand their skills in other areas critical to chronic disease prevention (SPD).

## Essential Activities

We identified 4 activities that were essential for a coordinated approach: 1) consistent communication and messaging, 2) evidence-based interventions, 3), supporting strong program infrastructure, and 4) strategic use of staff.

“Consistent communication and messaging” represents state health departments’ efforts in communicating more holistically about chronic diseases in ways that are easier for outside audiences to understand. Many state health departments created an internal communications unit or function that provided support and guidance to programs on branding and message consistency. A good example is the strategic release of data by state health departments. Several states created coordinated chronic disease infographics that showed the connections across risk factors and chronic diseases, rather than lengthy burden reports of a single disease (http://dphhs.mt.gov/publichealth/chronicdisease and http://www.healthy.ohio.gov/~/media/HealthyOhio/ASSETS/Files/creating%20healthy%20communities/CHC%202013%20Infographic.pdf).

Brownson et al define “evidence-based public health” as a process that includes, among other elements, “making decisions on the basis of the best available scientific evidence” (11). State health departments, in developing their vision for chronic disease prevention and the chronic disease plans, ensured that evidence-based strategies guided their decision-making processes.

There’s a focus on best practices. We don’t spend money or time on things that are not best practices or evidence-based. The creation of specialized branches and units in communications, data analysis, and policy and health systems work led to a greater emphasis on data-driven decision-making and evidence-based planning (SPD).

In addition, an emphasis was renewed on the underlying social determinants that drive poor health outcomes, better positioning state health departments to focus on these issues and promote them in and outside their agencies.

Our efforts to focus on social determinants of health have filtered up to the governor’s office. As part of the governor’s initiative, one of the benchmarks of success is increasing high school grad[uation] rates. They have education, tourism, labor, and others at the table and are recognizing that those areas are part of health (CDDI).

If we’re trying to get to underlying issues, a coordinated approach gives us better leverage compared to disease-specific work. If we have less money and are more siloed, we tend to drift to higher SES [socioeconomic status] groups [with our programmatic efforts] in order to show impact quickly (CDDI).

When states build critical infrastructure from a single funding source (eg, a CDC award, state funding, foundation grant, or Master Settlement Agreement), they have setbacks in prevention efforts when the funding ends (12). Many find themselves rebuilding the same structures and prevention efforts a few years later when state priorities shift or when they receive a new federal award. States examined their structure and organization as a result of the CCDP funding and made various changes: 1) workgroups for critical functions, 2) reclassification of key positions, or 3) reorganization of the chronic disease unit. These efforts helped to stabilize critical long-term infrastructure so that changes in funding are more easily weathered and critical functions can be sustained. “The cross-cutting alignment of staff works. When someone in the health systems-cancer program leaves, we have staff in health systems that can cover it” (CDDI).

The CCDP program created the opportunity for state health departments to be proactive about building the competencies of their staff by implementing self-assessments related to public health competencies, as well as individual and bureau-level training plans. States assessed the readiness of their staff to meet new public health challenges and addressed any gaps by linking individual training plans to core public health competencies with an eye toward succession planning.

## Discussion

The conceptual model for chronic disease coordination outlines key components and strategies that can be used to establish linkages between and among categorical programs and communicate the important benefits of a coordinated approach. States have made progress in areas such as developing a focused agenda at the state level, which results in state health departments’ getting “seats” at “bigger tables” with major health care systems, developing stable infrastructure that does not depend on a sole source of funding, and managing resources in a way that deepens skill sets of staff and more efficiently spreads expertise across the entire chronic disease unit. And according to meeting notes between NCCDPHP leadership and state chronic disease directors, NCCDPHP’s 4 domains have been adopted widely by states as a way to frame the work that they do.

Although these benefits better prepare state health departments for the demands on public health in the 21st century, states also recognize the difficulties and costs associated with this change. State health departments continue to grapple with the confines of funding agreements, the relationship between siloed funding categories, and a concern about the loss of disease-specific champions and staff.

**Figure 2:**
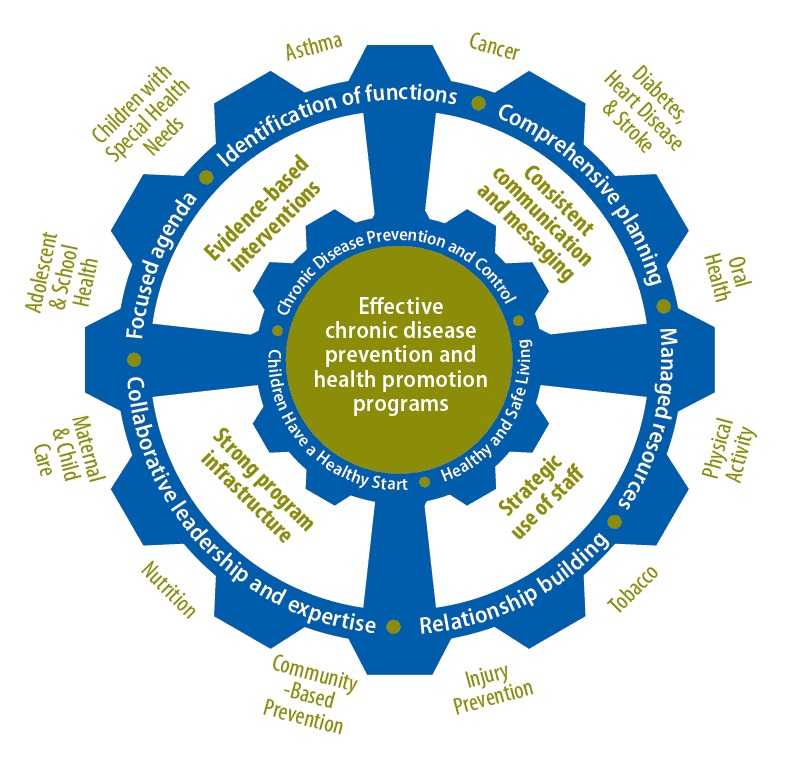
Conceptual model for chronic disease coordination — Maine State Department of Health, 2015.

The model takes into account these concerns by leaving room for the clear identification of disease-specific programs (Figure 2). The figure shows a potential conceptual model for coordinated chronic disease efforts in Maine’s state health department. It includes disease-specific programs located in the Maine chronic disease unit and its organizing framework: Children Have a Healthy Start, Healthy and Safe Living, and Chronic Disease Prevention and Control. The ability to customize the model according to a state health department’s context is a unique feature that will make the model more applicable to states’ priorities.

This article is subject to a few limitations. The conceptual model was based on the experiences of state health departments that had not only achieved the objectives of the CCDP program but also sustained these changes. Thus, states that had challenges may find the model to be less representative. An important test of the model’s validity will be the use of this model to replicate success in coordination, which we plan to review in a subsequent publication. In addition, these findings represent the experience of the CCDP program and may not reflect more recent experiences, such as the major shifts in NCCDPHP funding to state health departments through the State Public Health Actions program in 2013.

Despite these limitations, this study contributes to the literature on chronic disease coordination. The elements of leadership and management and essential activities can be used to support training and technical assistance. The model’s built-in flexibility allows state health departments as well as local health departments and other organizations to replicate efforts in coordination, providing the opportunity to further validate these findings. Finally, the model can be used as a framework for evaluating a coordinated approach to chronic disease prevention and control. CDC plans to disseminate the model through various media, including the creation of a web-based interactive version that can be customized according to a state or local context. More study is needed on how these elements of chronic disease coordination interact with one another to create more efficient and effective chronic disease prevention and control programs.
