# ERRATUM — Vol. 13, March 31 Release

**DOI:** 10.5888/pcd13.150509e

**Published:** 2016-04-14

In the article “A Customizable Model for Chronic Disease Coordination: Lessons Learned From the Coordinated Chronic Disease Program,” 2 figures were omitted because of a technical error.

The correction was made to our website on March 31, 2016, and appears online at http://www.cdc.gov/pcd/issues/2016/15_0509.htm. We regret any confusion or inconvenience this error may have caused.

